# Retrospective cohort study of pregnancy outcomes in hidradenitis suppurativa

**DOI:** 10.1111/bjd.19155

**Published:** 2020-06-10

**Authors:** A.B. Lyon, A. Peacock, S.A. McKenzie, G. Jacobsen, H.B. Naik, V.Y. Shi, I.H. Hamzavi, J.L. Hsiao

**Affiliations:** 1 Department of Dermatology, Henry Ford Hospital, Detroit, MI, USA; 2 Department of Internal Medicine, St Mary Mercy Hospital, Livonia, MI, USA; 3 Department of Dermatology, University of California Los Angeles, Los Angeles, CA, USA; 4 Department of Dermatology, University of California San Francisco, San Francisco, CA, USA; 5 Department of Dermatology, University of Arizona, Tucson, AZ, USA

Dear Editor, Hidradenitis suppurativa (HS) disproportionately affects women of childbearing age; however, there has been a paucity of literature in the field of HS and pregnancy.^1^ The objective of this study was to examine pregnancy complications, pregnancy outcomes and neonatal outcomes in patients with HS.

This retrospective cohort study was approved by the institutional review board at Henry Ford Hospital. All patients with a diagnosis of HS who became pregnant between January 2008 and December 2018 were identified using International Classification of Diseases 9th and 10th Revision codes for HS (705·83, L73·2), pregnancy (V22·1, Z34·00, Z34·01, Z34·02, Z34·03, Z34·90), active labour (649·8, O75·82), vaginal delivery (650, O80, Z37·0), caesarean delivery (669·7, O82·9, O82·8, O82·2, O82·1), stillbirth (V27·1, Z37·1) and miscarriage (634·91, O03·9). Demographics, comorbidities, pregnancy outcomes and neonatal outcomes were collected through manual chart review of the electronic medical records. Data for Hurley stage of HS were extracted from clinic notes at the beginning of each pregnancy. A board-certified dermatologist (J.L.H.) reviewed the physical exam findings of each chart to determine the Hurley stage for each patient. Patient charts were reviewed from the beginning of their pregnancy through to 6 months postpregnancy.

Within-mother independence was not assumed for mothers with multiple pregnancies. Generalized estimating equation modelling was used to evaluate the following: (i) associations of miscarriage status with race, baseline obesity and Hurley stage; (ii) associations of breastfeeding status with presence of breast HS and severe HS (Hurley stage 3); (iii) likelihood of caesarean section (C-section) based on the presence of groin or vulvar HS lesions; and (iv) associations of baseline Hurley stage with live birth, C-section, gestational hypertension, pre-eclampsia, pregnancy complication and neonatal complication. In addition, HS cohort percentages were compared with corresponding general US population percentages using one-sample χ^2^-tests. P-values < 0·05 were considered statistically significant. The study sample size resulted in a power of 0·80 to detect modest outcome differences of 12–18% for individual patient characteristics. All analyses were performed using SAS version 9·4 (SAS Institute, Cary, NC, USA).

In total 202 pregnancies in 127 patients with HS were included (Table 1). Tobacco and marijuana use was continued throughout 13·4% (n = 27) and 13·9% (n = 28) of pregnancies, respectively. Likelihood of miscarriage was not significantly associated with race, obesity at baseline, or Hurley stage. Presence of vulvar or groin HS lesions was not associated with a higher likelihood of C-section.

Baseline HS severity was not significantly associated with rate of live birth (P = 0·59), likelihood of C-section (P = 0·66), or increased risk of gestational hypertension (P = 0·080), preeclampsia (P = 0·53), pregnancy complication (P = 0·92) or neonatal complication (P = 0·23). Having HS lesions on the breast was significantly associated with not breastfeeding (P = 0·004). A significantly higher proportion of patients with Hurley stage 3 did not breastfeed compared with Hurley stage 1 or 2 (P = 0·039).

In over 10% of pregnancies in this cohort, patients continued to use tobacco and marijuana during pregnancy; therefore, strongly counselling patients with HS regarding cessation of smoking and recreational drug use during pregnancy is warranted. Additionally, a significantly higher proportion of patients with HS breast lesions did not breastfeed compared with those without HS breast lesions. This highlights the importance of anticipatory counselling regarding breastfeeding in patients with breast lesions.

Prior studies on other inflammatory diseases such as rheumatoid arthritis and systemic lupus erythematosus have shown that the disease influences rates of preterm birth and pregnancy loss.^2^ No statistically significant differences were detected when comparing our study cohort with the general population of women in the US in terms of the rates of miscarriage,^3^ C-section,^4^ premature baby,^4^ stillbirth^5^ and perinatal mortality.^5^ In addition, baseline HS disease severity was not significantly associated with poorer pregnancy or neonatal outcomes. Thus, our findings on pregnancy outcomes can help providers counsel and reassure concerned pregnant patients with HS regarding pregnancy outcomes. In contrast, compared with rates of gestational diabetes mellitus,^6^ gestational hypertension^7^ and pre-eclampsia^8^ in the US general population, our study cohort had a higher than average rate for each condition, with statistical significance detected for gestational hypertension (P = 0·022) and pre-eclampsia (P = 0·017). Therefore, screening for these conditions among pregnant patients with HS is essential.

Study limitations include: that it took place at a single academic centre, was retrospective and contained missing data. Lack of systematic follow-up of neonatal charts could have resulted in underestimation of the perinatal mortality rate. Only univariate analysis was conducted; thus, potential confounders have not been controlled for.

In summary, our study suggests that HS does not portend an increased risk of poor pregnancy or neonatal outcomes. Large, prospective pregnancy registries are needed to collect data on maternal and neonatal outcomes in patients with HS.

## Figures and Tables

**Table 1:** Hidradenitis suppurativa (HS) pregnancy characteristics and outcomes, and comparisons with the general US population

Pregnancy characteristics and outcomes (202 total pregnancies)			Value

Age at time of pregnancy (years), mean ± SD (IQR) (n = 202)			25·9 ± 5·03 (22–28)
Patient’s race (n = 199): black, white, other			171 (85·9), 25 (12·6), 3 (1·5)
Obese (BMI ≥ 30 kg m^−2^) at baseline (n = 163): yes, no			102 (62·6), 61 (37·4)
Hurley stage (n = 191): 1, 2, 3			103 (53·9), 70 (36·6), 18 (9·4)
Location of HS (n = 201): axillae, groin, breasts, buttocks, abdomen, chest			147 (73·1), 104 (51·7), 48 (23·9), 36 (17·9), 16 (8·0), 12 (6·0)
Gestational diabetes mellitus (n = 194): yes, no			18 (9·3), 176 (90·7)
Gestational hypertension (n = 194): yes, no			29 (14·9), 165 (85·1)
Pregnancy complication (n = 200): miscarriage, pre-eclampsia, preterm labour, other,^a^ none			28 (14·0), 16 (8·0), 4 (2·0), 4 (2·0), 148 (74·0)
Live birth (n = 202): yes, no			161 (79·7), 41 (20·3)
Pregnancy term (n = 160): preterm (< 37 weeks), term (37·42 weeks)			9 (5·6), 151 (94·4)
Method of delivery (n = 157): vaginal delivery, C-section for any reason			105 (66·9), 52 (33·1)
Method of feeding (n = 134): breast, bottle			102 (76·1), 32 (23·9)
Neonatal complications (n = 148):^b^ yes, no			19 (12·8), 129 (87·2)
Pregnancy complications: comparison of the HS cohort with the general US population			
	HS cohort	General US population^c^	Comparison P-value
Gestational diabetes mellitus	9·3%	6–8%	0·21
Gestational hypertension	14·9%	10%	0·022*
Miscarriage	14·0%	10–15%	0·52
Pre-eclampsia	8·0%	3–6%	0·017*
Premature baby	5·6%	9·9%	0·070
C-section	33·1%	32%	0·76
Stillbirth	0%	1%	0·15
Perinatal mortality	1·2%	3·1%	0·17

The data are presented as n (%) unless stated otherwise. The number of available data are provided. BMI, body mass index; IQR, interquartile range.

a Other includes placental abruption (2), severe anaemia requiring transfusion (1) and chorioamnionitis (1).

b Neonatal complication includes shoulder dystocia (4), premature requiring neonatal intensive care unit (NICU) stay (4), death (2), fetal intolerance (2), respiratory distress requiring NICU stay (1), nonreassuring fetal heart tones requiring NICU stay (1), bilateral hydronephrosis (1), gastroenteritis (1) and unknown (3).

c When the US population involves a range, the midpoint was used for comparison purposes.

* Statistically significant, P < 0·05.
